# Antiproliferative Effect of Indole Phytoalexins

**DOI:** 10.3390/molecules21121626

**Published:** 2016-11-26

**Authors:** Martina Chripkova, Frantisek Zigo, Jan Mojzis

**Affiliations:** 1 Department of Pharmacology, Faculty of Medicine, P.J. Šafárik University, 040 11 Košice, Slovakia; chripkova.martina@gmail.com; 2 Department of Human and Clinical Pharmacology, University of Veterinary Medicine and Pharmacy, 040 11 Košice, Slovakia; 3 Department of Animal Breeding, University of Veterinary Medicine and Pharmacy, 040 11 Košice, Slovakia; frantisek.zigo@uvlf.sk

**Keywords:** indole phytoalexins, crucifers, brassinin, antiproliferative, cancer

## Abstract

Indole phytoalexins from crucifers have been shown to exhibit significant anti-cancer, chemopreventive, and antiproliferative activity. Phytoalexins are natural low molecular antimicrobial compounds that are synthesized and accumulated in plants after their exposure to pathogenic microorganisms. Most interestingly, crucifers appear to be the only plant family producing sulfur-containing indole phytoalexins. The mechanisms underlying its anti-cancer properties are unknown. Isolation from cruciferous plants does not provide sufficient quantities of indole phytoalexins and, for biological screening, they are usually obtainable through synthesis. Understanding the molecular mechanism of the action of these substances and their structure-activity relationships is quite important in the development of new analogs with a more favorable profile of biological activities. In this review, we present the key features of indole phytoalexins, mainly their antiproliferative ativities.

## 1. Introduction

Substances of plant origin have a significant position among the drugs used for cancer treatment. Information about the healing effects of extracts from various plants can be found in Chinese, Indian, as well as ancient literature [1]. The tradition in their use and claimed successes in anti-tumor therapy are an inspiration for a more detailed study. Modern methods in biochemistry and pharmacology allow their chemical identification and exact testing.

The discovery of the anti-cancer effects of foods rich in vegetables from the *Cruciferae* (*Brassicaceace*) family led to an increased interest in the natural substances contained therein [2]. Their frequent consumption, along with other kinds of vegetables and fruits, can significantly reduce the risk of cancer and prolong a patient’s life [3]. Although cruciferous vegetables contain many compounds with anti-cancer properties, they are unique because of the high content of sulfur-containing phytochemicals, the glucosinolates [4]. After metabolic degradation, glucosinolates are converted to a variety of products, from which the isothiocyanates play probably the most important role in cancer chemoprevention [5]. During the last 20 years, multiple mechanisms of their anti-cancer action have been recognized, including modulation of carcinogen metabolism [6,7,8], induction of apoptosis and cell cycle arrest, inhibition of neovascularization [9,10], inhibition of cancer cell migration [11,12], as well as the blocking of signaling pathways associated with malignant transformation or cell survival [13,14,15].

Furthermore, a special group of substances in cruciferous plants are the indole phytoalexins. Indole phytoalexins have been reviewed with regard to their isolation, occurrence, synthesis, biosynthesis, biotransformation, and role in plant defenses [16,17,18,19,20,21]. These substances show a wide range of biological effects, including antiproliferative [22,23,24], antifungal [25], antiprotozoal [26], chemopreventive [27], and anti-cancer effects [28]. While a variety of possible molecular mechanisms have been proposed to explain this activity, none has been directly validated in vivo.

## 2. Occurrence, Structure, and Biological Activity of Indole Phytoalexins

Phytoalexins are antimicrobial secondary metabolites with a low molecular weight produced *de novo* by plants after exposure to biological (bacteria, fungi, viruses), physical (UV radiation, heat shock, injury), or chemical (heavy metals) stress [20,29]. They include a number of different types of substances that are species-specific, such as terpenoids, alkaloids, flavonoids, and the like. The name of phytoalexins comes from their function of defending the plant organism, as it is derived from the Greek words phyton–plant, alexos–defend. They are not located in the tissues of healthy plants. The synthesis of phytoalexins is triggered by specific substances, so-called elicitors, in plants. These substances initiate the defense response of a plant after it is attacked by a pathogen. Phytoalexins appear in an infected plant several hours or days after such an attack [30,31,32]. These substances are produced by plants in small amounts, and isolation of them from plants is difficult. The introduction of the chemical synthesis and their analogs provided the appropriate quantities necessary for an evaluation of their environmental function and biological activity [21].

Phytoalexins were first described by Müller and Börger in 1940 as antifungal substances produced by *Solanum tuberosum* after being attacked by the fungus *Phytophora infestans* [33]. The first phytoalexin to be isolated and chemically characterized was (+)-pisatin. Pisatin was obtained from *Pisum sativum* (pea) in a small concentration after the plant was infected by the *Ascochyta pisi* pathogen [34].

The structure of phytoalexins depends on the type of plant that synthesizes them and partially on the elicitor inducing their synthesis. Isoflavones prevail in plants of the *Fabaceae* family, sesquiterpenoids in the *Solanaceae* family, diterpenes in the *Poaceae* family, and for plants of the *Brassicaceae* family it is a characteristic that it is the only family that produces indole phytoalexins containing sulphurous phytoalexins [16,35].

**Figure 1:**
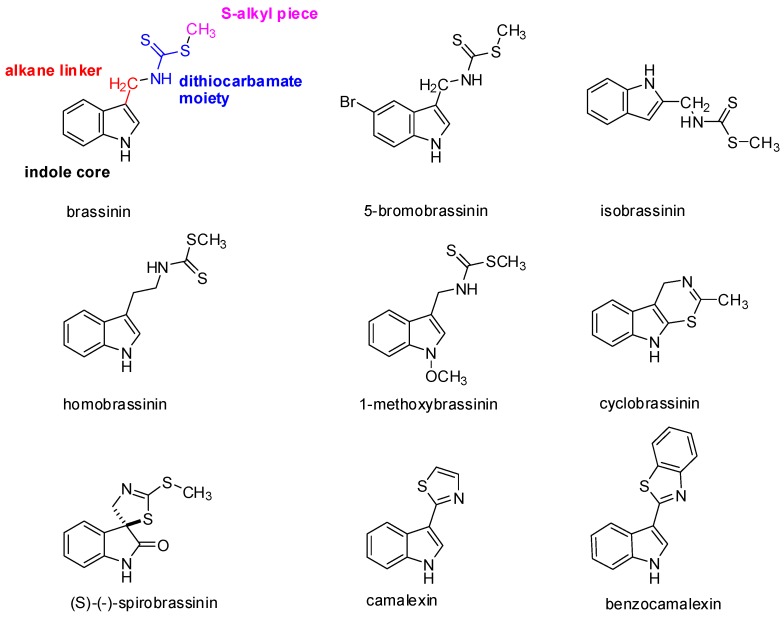
Molecular structures of the representative indole phytoalexins and their derivatives.

At present, 44 different species of indole phytoalexins are known. Some of them are produced by more than one plant species and may be elicited by a number of pathogens or abiotic factors [21]. What is interesting concerning the indole phytoalexins’ structure is the unique connection of the indole nucleus with the side chain or a heterocycle containing nitrogen and sulfur atoms [36]. The side chain is most often in the –CH_2_– group in position 3 of the indole nucleus. A heterocycle may be joined by condensation (cyclobrassinin), by a single bond (camalexin) or through a spiroatom (spirobrassinin) (Figure 1). Methyl-1-methoxy-indole-3-carboxylate has a carbonyl group (–C=O) at position 3 of the indole nucleus, discovered as the first of two phytoalexins of cruciferous plants that do not contain sulfur. It was isolated from wasabi, and it is assumed that its presence in this plant results in the crusting resistance caused by the fungus *Phoma lingam*. Brasicanal A, B, and C belong to the indole phytoalexins having an aldehyde group (–CH=O) at position 3 of the indole nucleus [18,37]. The bifunctional structural characteristic of natural brassinin, i.e., the position of both the indole nuclei and the dithiocarbamoyl aminomethyl moiety, is similar to that of the chemopreventive agents, such as indole-3-carbinol and benzyl isothiocyanate [38]. As was mentioned above, isothiocyanates and indoles derived from the hydrolysis of glucosinolates, such as sulforaphane and indole-3-carbinol, have been implicated in a variety of anticarcinogenic mechanisms [4]. With the presence of both components, a positive biological effect can be achieved. An aliphatic analog of brassinin, (±)-4-methylsulfinyl-1-(*S*-methyldithiocarbamyl)-butane (Sulforamate), has structural similarities to sulforaphane [38].

The biosynthetic pathway of indole phytoalexins begins with the conversion of tryptophan to indolyl-3-acetohydroxamic acid and then opens into a number of metabolic pathways, including indolyl glucosinolates, brassinins, and camalexin [39]. Brassinin is a biosynthetic precursor of several other phytoalexins [35].

Brassinin, cyclobrassinin, and 1-methoxybrassinin (Figure 1) are indole phytoalexins first isolated from Chinese cabbage which had been infected by the *Pseudomonas cichorii* bacterium [36]. Cyclobrassinin is a natural product of oxidative brassinin cyclization [27], while 1-methoxybrassinin is 1-methoxyindol alkaloid with a methoxy group bound to the indole nitrogen atom. Other typical representatives of 1-methoxyindol phytoalexins are 1-methoxybrassitin, also isolated from Chinese cabbage; 1-methoxybrasenin A and 1-methoxybrasenin B of cabbage; (*R*)-(+)-1-methoxyspirobrassinin isolated from kohlrabi; (2*R*,3*R*)-(−)-1-methoxyspirobrassinol methyl ether isolated from the Japanese radish; and sinalbin B from white mustard [17,18]. Later, arvelexin, located in *Thlaspi arvense* (field pennycress), was described [40]. Isalexin, brassicanate, and rutalexin were isolated from *Brassica napus*, ssp. *rapifera* (rutabaga) after abiotic elicitation through UV light or after being infected by the pathogenic fungus *Rhizoctonia solani* [25]. Erucalexin was obtained from *Erucastrum gallicum* (common dogmustard). The presence of caulilexin, caulilexin B, and caulilexin C was discovered in cauliflower [41]. *Thellungiella salsuginea* (saltwater cress) produces wasalexin A and B. In addition to wasalexins, this plant also produces the phytoalexins 1-methoxybrassenin B and rapalexin A [42]. Brussels sprouts were shown to produce a unique thiolcarbamate, brussalexin A. It is the first naturally occurring thiolcarbamate in which the sulfur atom is attached to the 3-methylindolyl moiety [43]. The phytoalexins 4-methoxycyclobrassinin and dehydrocyclobrassinin were isolated for the first time from canola roots infected with the biotroph *Plasmodiophora brassicae* (clubroot disease) [44].

(*S*)-(−)-Spirobrassinin (Figure 1) was isolated in 1987 from Japanese radish [45]. Spirobrassinin resembles other anticarcinogenic substances in its structure, such as pentacyclic oxindole alkaloids found in *Uncaria tomentosa* (cat’s claw) from the Andean region and Peru, a plant used in folk medicine as an anti-cancer and anti-inflammatory substance as well as a contraceptive [46]. These alkaloids have an antiproliferative effect on HL-60 and U-937 leukemic cell lines, without inhibiting the growth of progenitor cells, with the highest activity observed for Uncarina F (IC_50_ = 21.7–29 μmol/L) [47].

Camalexin (Figure 1) has a remarkable position among the indole phytoalexins in that it is produced by the *Arabidopsis thaliana* plant (Arabidopsis willow) after being infected by the *Alternaria brassicicola* fungus and *Pseudomonas syringae* bacterium. The presence of this substance has also been demonstrated in the plants *Capsella bursa-pastoris* (shepherd’s pocket) and *Camelina sativa* (gold-of-pleasure). The genetic data show that camalexin is synthesized from tryptophan. The reaction is catalyzed by two kinds of P450cytochrome (CYP79B2 and CYP71B15) [48]. Biosynthesis is localized at the site of pathogen infection and takes place in the endoplasmic reticulum [49]. This phytoalexin has a cytostatic effect on the *Trypanosoma cruzi* pathogenic flagellate [26].

What is remarkable in indole phytoalexins is the presence of a dithiocarbamate group (NH-CS-SR) (Figure 1), which is part of some organic fungicides [50]. Dithiocarbamates are distinguished through strong antioxidant and antitumor effects. It has been confirmed that the dithiocarbamate side chain is very crucial for the anti-cancer activity [51]. Reactive metabolites of certain dithiocarbamates (proline-dithiocarbamate, diethyldithiocarbamate) induce the expression of p21KIP1/CIP1 in the p53 dependent pathway, leading to the cessation of the cell cycle in a G1/S HepG2 cell line. Furthermore, they affect the phosphorylation of cyclin E, the cyclin of dependent kinase inhibition 2, and cyclin E degradation in these cells during apoptosis. They also cause a decrease of Bcl-2 anti-apoptotic proteins and an increase in the level of p53 protein. 4(3*H*)-Quinazolinone dithiocarbamate exhibited anti-cancer activity against human myelogenous leukaemia cells [52,53].

Duan et al., [54] reported the synthesis of a series of novel 1,2,3-triazole-dithiocarbamate hybrids and evaluated them for anti-cancer activity against several human tumor cell lines (MGC-803, MCF-7, PC-3, EC-109). Another study reported novel dithiocarbamte derivatives where benzimidazole replaced brassinin in the indole moiety. Their chemotherapeutic activity was evaluated. This docking study revealed that benzimidazoledithiocarbamate derivatives are more selective for anti-cancer activity than antimicrobial activity [55]. Some dithiocarbamates act by modulating the responses of heat shock proteins (HSP—they synthesize in the cell as a response to the influence of any stress) or by inhibiting the activity of the NF-κB transcription factor [19]. In the case of tumor cells, the activation of NF-κB plays a role in the protection against apoptosis induced by e.g., the action of TNF-α, ionizing radiation, or other inducements. The activated signaling pathway of NF-κB inhibits the apoptotic potential of chemotherapeutic agents and thereby contributes to the resistance of cancer cells to these agents. Therefore, it follows that the substance inhibiting the signaling pathway may be used to overcome the drug resistance of tumor cells [56]. NF-κB also regulates the expression of a large number of genes that play an important role in the non-adaptive (innate) immune response [57]. In connection to this fact, the anti-inflammatory effects of arvelexin, which inhibits the activation of NF-κB in macrophages with a subsequent decrease of the expression of pro-inflammatory inducible enzymes (iNOS, COX-2) and cytokines (TNF-a, IL-6 and IL-1b), were demonstrated [58].

## 3. The Antiproliferative Effect of Naturally Occurring Indole Phytoalexins

The ability of indole phytoalexins to inhibit the growth of cells was tested in vitro in a number of cancer cell lines. The mechanism of the antiproliferative effect of these substances is still unclear. Available data indicate that the antiproliferative activity of indole phytoalexins is more a result of modulating the activity of transcription factors regulating the cell cycle, cell differentiation, and apoptosis than a direct interaction with DNA [19,59]. The possible anti-cancer effects of representative indole phytoalexins and their derivatives are summarized in Table 1.

The antiproliferative effect of brassinin, spirobrassinin, and cyclobrassinin was tested in a (B16) mouse melanoma and (L1210) leukemia cancer cell line. Brassinin showed the highest inhibitory effect in that, at a 100 μmol/L concentration, it reduced the cell growth by 35% and at 10 μmol/L concentration by 15% (L1210) and 9% (B16), respectively, after 24 h of incubation. Spirobrassinin was less effective; a reduction of the number of cells occurred only at a concentration of 100 μmol/L (about 13%). Cyclobrassinin did not show antiproliferative activity [59].

Significant chemopreventive activity was also recorded in these substances in a DMBA model (7,12-dimethylbenzathracene) of induced mammary gland carcinogenesis in mice. Brassinin inhibited the formation of pre-neoplastic lesions of the mammary gland by 73%, cyclobrassinin by 90.9%, and spirobrassinin by 76% at 10 μmol/L concentration. The mechanism of the chemopreventive effect is unknown, but probably the induction of phase II detoxification enzymes occurs. Regarding the inhibition of tumor growth, these substances may have a chemopreventive effect in the initiation and promotional phases of carcinogenesis (Table 1) [27,38,60].

Cyclobrassinin, brassilexin, and their synthetic analogs (homocyclobrassinin and 5-methoxybrassilexin) caused growth inhibition of the KB cell line (epidermoid carcinoma), while the highest efficiency was observed in brassilexin (IC_50_ 8 μg/mL). Brassilexin had the same values of IC_50_ for human KB carcinoma and normal monkey kidney cells, which indicates a lack of selectivity for cancer cells [61].

Brassinin and its derivatives are inhibitors of indoleamine 2,3-dioxygenase (IDO), which is a new target in cancer immunotherapy. IDO is an extrahepatic enzyme that catalyzes the initial and rate-limiting step in the degradation of tryptophan along the kynurenine pathway that leads to the biosynthesis of nicotinamide adenine dinucleotide (NAD^+^) [62,63,64]. The degradation of tryptophan reduces the immune response to tumor cells. Therefore, blocking IDO could lead to greater efficiency in tumor immunotherapy [65]. The role of the inhibition of IDO in the antitumor mechanism of the bioavailable analog of brassinin (5-bromobrassinin (Figure 1)) was confirmed in vivo, where this compound suppressed growth of B16-F10 melanoma xenografts in C57BL/6 mice but not in athymic NCr-nu/nu and IDO knock-out mice (Table 1) [28].

Gaspari et al. [66] undertook a structure-activity relationship study of brassinin with the goal of obtaining a more potent IDO inhibitor. They divided the brassinin structure into four components: the indole core, the alkane linker, the dithiocarbamate moiety, and the S-alkyl piece (Figure 1). The study showed that replacement of the indole moiety with other aromatic rings retained the activity, whereas an increase in the length of alkyl chain increased the potency of compounds, but upon replacement of the dithiocarbamate side chain with any other group, the compounds exhibited very little or no activity at all.

Brassinin has been reported to induce G1 phase arrest through the increase of p21 and p27 by inhibition of the phosphatidylinositol 3-kinase signaling pathway in colorectal cancer cells [67]. The latest data suggests possible brassinin interference with the PI3K/Akt/mTOR/S6K1 signaling pathway [68]. Regulation of the mTOR (mammalian target of rapamycine) protein kinase plays an important role in the cellular metabolism of proliferation and angiogenesis. It is an attractive therapeutic target because it is a key point at which a number of signaling pathways converge. The activation of the PI3K/Akt/mTOR/S6K1 signaling pathway is closely linked with the development of prostate cancer, its metastasis, and angiogenesis [69]. The ability of brassinin to inhibit this cascade is pre-tagging it as a potential candidate for the treatment and prevention of prostate cancer [68]. Brassinin can inhibit the constitutive and inducible STAT3 (Signal transducer and activator of transcription 3) signaling pathway, thereby attenuating tumor growth (Table 1) [70]. STAT are proteins that regulate gene expression by affecting transcription. They are part of the signal transduction pathway of many growth factors and cytokines and are activated by phosphorylation of tyrosine and serine residues by upstream kinases [71]. Constitutive activation of STAT3 has been reported in many types of malignancies, such as myeloma, head and neck cancer, breast cancer, prostate cancer, and non-small cell lung cancers (NSCLC) [72,73,74,75,76,77]. There is evidence showing that inhibition of STAT3 leads to cessation of tumor cell growth and apoptosis. Brassinin suppressed STAT3 activation through the modulation of two groups of signaling proteins known to inactivate STAT proteins, the protein inhibitors of activated STAT (PIAS) and the suppressors of cytokine signaling (SOCS). In addition, brassinin enhanced the antitumor effects of paclitaxel, a chemotherapeutic drug used extensively to treat NSCLC (non-small cell lung cancer) patients [70].

Kim et al. [78] analyzed the potential synergistic anti-tumor effects of brassinin combined with capsaicin on prostate cancer PC-3 cells (Table 1). Capsaicin, an alkaloid derived from the chilli pepper, has been shown to promote cell death in a variety of tumor cells [79]. After treatment with brassinin and capsaicin at various concentrations, the synergistic cytotoxic effect of PC-3 cells was observed [78].

In our studies, we examined the antiproliferation effects of 1-methoxybrassinin [24] and homobrassinin (Table 1), (Figure 1) [80]. We found the redistribution of the cell contents into the G2/M phase after just 24 hours of incubation with homobrassinin. The accumulation of cells in the G2/M phase could indicate a possible interaction of this substance with tubulins, which are involved in the construction of the spindle apparatus. This theory is supported by the results of the study by Smith et al. [81], in which they reported that the degradation products of glucosinolates, substances biogenetically related to indole phytoalexins, caused the condensation of α-tubulin and subsequent blocking of the mitotic phase in colorectal cancer cells (Caco-2). The results of cell cycle analysis led us to monitor the expression of selected genes involving the formation of microtubules. We found changes in the expression of tubulin subunits after exposure to homobrassinin in the form of a reduced expression of β5-tubulin and increased expression of α-tubulin. The ability of the studied substances to induce the apoptosis of Caco-2 cells was associated with changes in the balance between pro- and anti-apoptotic representatives of proteins of the Bcl-2 family and caspase-3 activation [80]. 1-methoxybrassinin showed significant antiproliferative effects on the Jurkat (human acute T lymphoblastic leukemia) cell line (IC_50_ 10 μmol/L). Cell cycle analysis showed a reduction in the number of cells in the S and G2/M phase of the cell cycle with an increased fraction of sub-G0/G1 DNA, which is considered a marker of apoptosis. After 72 h of the incubation of Jurkat cells with 1-methoxybrassinin, the amount of this fraction increased to more than 90% [22]. The increase in ROS levels, reduction in the mitochondrial membrane potential levels, and decrease of GSH in the Caco-2 cells after treatment with 1-methoxybrassinin probably also contribute to the triggering of the apoptotic cascade. The potential of this substance to cause GSH depletion in tumor cells could be used to increase their sensitivity to chemotherapeutic drugs [24]. The significant potentiation of vincristine cytotoxicity to U-87 MG (human glioblastoma astrocytoma) cells by brassinin, spirobrassinin, 1-methoxyspirobrassinin, and 1-methoxyspirobrassinol, as well as drug-like characters of these compounds, suggest the possibility of their future role in combination chemotherapy [82].

While several reports showed the antiproliferative effect of cruciferous phytoalexins, a study by Mezencev et al. revealed a contradictory effect. While spirobrassinin and 1-methoxyspirobrassinol methyl ether reduced the growth of MCF-7 (breast cancer cell line, estrogen receptor positive) and Caco-2, brassinin, 1-methoxyspirobrassinol, and 1-methoxyspirobrassinin in contrast stimulated the proliferation of these cells. All tested substances inhibited the growth of the MDA-MB-231 (breast cancer cell line). It can therefore be assumed that MCF-7 growth stimulation may be caused by the partial estrogen-receptor agonism of these indole phytoalexins and their metabolites [82].

**Figure 2:**
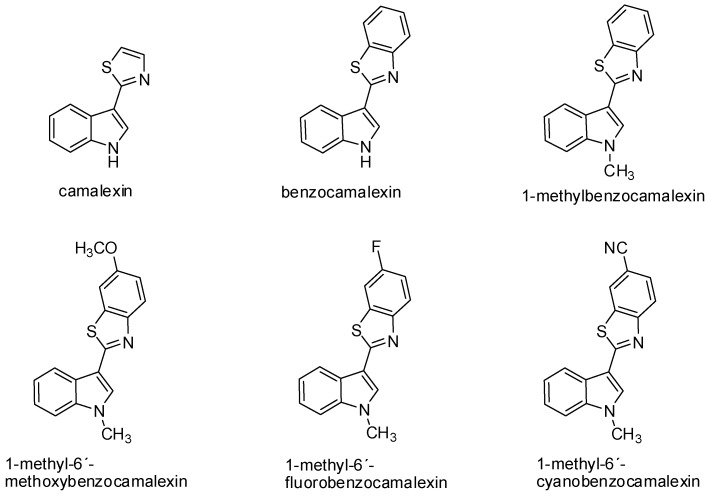
Chemical structure of camalexin and its synthetic analogs.

Camalexin has demonstrated antiproliferative activity on SKBr3 (human breast carcinoma cell line) with the increased expression of topoisomerase IIα. The inhibition of tumor cell growth induced by camalexin (IC_50_ 2.7 μmol/L) was even more evident when compared to conventional cytostatic agents, such as melphalan (IC_50_ 13.0 μmol/L) and cisplatin (IC_50_ 7.4 μmol/L) [83]. The mechanism of the cytotoxic effect of camalexin on Jurkat cells can be compared to the action of the ATO drug (arsenic trioxide) used for the therapy of relapse and resistant acute promyelocytic leukemia [84,85]. Data from recent years suggest that camalexin causes the accumulation of reactive forms of oxygen in tumor cells, resulting in the formation of oxidative stress, the activation of caspases, and induction of apoptosis. This effect was shown in the metastatic prostate cancer cell line and leukemia cell line. Inhibition of the growth of prostate cancer cells may be associated with a change of expression and activity of the cathepsin lysosomal enzyme in these cells due to camalexin influence. This hypothesis was also confirmed by an experiment with pepstatin A, an inhibitor of cathepsins activity, which blocked the cytotoxic effect of camalexin. Cathepsins are secreted into the cytosol during the initiation of apoptosis. Various incentives—such as oxidative stress, TNF-α, and p53—can increase lysosomal membrane permeabilization, thus triggering the translocation of these enzymes into the cytosol. Affecting the activity of lysosomal proteases, such as cathepsin, represents a great potential—particularly in the treatment of metastatic prostate cancer [86]. The advantage of camalexin, as well as its derivatives is its minimal cytotoxic effect on non-tumor cells (Table 1) [23,85]. Furthermore, in our study we have found structure-activity relationship. The fusion of benzene with thiazole ring of camalexin significantly enhances its cytotoxicity. On the other hand, further modulation of chemical structure (e.g., methylation of benzocamalexin) resulted in decreased antiproliferative activity and neither addition of methoxy-, fluoro-, nor cyano-group increased it (Figure 2) [85].

## 4. Antiproliferative Effect of Synthetic Derivatives of Indole Phytoalexins

Indole phytoalexins represent a natural template for the synthesis of several substituted derivatives in order to find more favorable antiproliferative and chemopreventive effects of these substances.

Glyoxylic analogs of natural phytoalexins, such as brassinin, brassitin, and some 1-methoxyindole phytoalexins, were synthesized. When comparing the antiproliferative activity of these derivatives to natural phytoalexins, it was shown that the most effective was an analog of glyoxylic 1-methoxybrassenin B (IC_50_ 3.3–66.1 μmol/L), which reduced the growth of cells in the most acute lymphoblastic leukemia line (CCRF-CEM) from a number of tested cancer cell lines (Jurkat, HeLa, MCF-7, MDA-MB231, A-549, CCRF-CEM) [87]. There are also several other studies on the anti-tumor effects of substances containing the indolyl glyoxylic group. Glyoxylic derivatives have been found as intermediates in the synthesis of anti-tumor indolocarbazole alkaloids. The indolyl glyoxylic group is part of the natural marine product, hyrtiosin B (isolated from the sea sponge *Hyrtios erecta*), which has shown in vitro antiproliferative activity against the KB cell line [88]. Synthetic indolyl glyoxylic amides have been identified as anti-tumor substances destabilizing the microtubules of cells, with indibulin as the most active derivative. This derivative was characterized as demonstrating in vitro activity against tumor SKOV3 (ovarian cancer), U87 (glioblastoma), and ASPC-1 (pancreas adenocarcinoma) cell lines [89].

Various synthetic 2-amino derivatives of spiroindoline phytoalexins have shown remarkable anti-tumor features. Through the introduction of a substituted phenylamino group into position 2 of the indole ring of 1-methoxyspirobrassinol methyl ether derivatives with a better anti-tumor effect than natural phytoalexins themselves were obtained. Some even achieved a better antiproliferative effect on cancer cell lines, such as cisplatin, etoposide, and doxorubicin [90]. Similarly, the 2-amino derivative of 1-methoxyspirobrassinol, *trans*-1-Boc-2-deoxy-2-(1-piperidinyl) spirobrassinol created an anti-tumor effect against small cell lung carcinoma, renal, ovarian, prostate carcinoma, and colorectal carcinoma and induced glutathione depletion in MCF-7 breast cancer cells [91]. The findings of these studies are useful for the suggestion of more amino analogs. By substituting the bis (2-chloroethyl) amino alkyl group into the above-mentioned amino derivatives of 1-methoxyspirobrassinol, the following compounds arose: *cis*- and *trans*-1-methoxy-2-deoxy-2 [*N*,*N*-bis(2-chloroethyl)amino] spirobrassinol. It follows that these synthetic analogs acquired the feature of alkylating substances to destabilize dsDNA and the capability of inducing the depletion of glutathione in tumor cells. Both compounds demonstrated in vitro antiproliferative activity against tumor cells of the ovarian adenocarcinoma and leukemia cell lines. Compared to the antitumor alkylating agent melphalan, the *cis*-amino derivative had a more notable antiproliferative effect on the ovarian adenocarcinoma cell line. Jurkat-M tumor cells (melphalan-resistant) showed less resistance against this amino compound [92].

In order to improve the anti-cancer activity of the natural phytoalexin cyclobrassinin, its new analogs with NR1R2 group instead of SCH3 were synthesized and evaluated. Several new analogs demonstrated higher antiproliferative potency than natural phytoalexin on at least one evaluated cancer cell line (Jurkat, MCF-7, MDA-MB-23, HeLa, CCRF-CEM, and A-549). The substance *N*-[1-(tert-Butoxycarbonyl)indol-3-yl]methyl-*N*′-phenylthiourea, which was found to be the most potent among all tested compounds on the MCF-7 cells, displays a potency very close to that of doxorubicin on these cells. Replacement of the 2-methylthio moiety of cyclobrassinin with an Ar-NH group resulted in a considerable increase in potency relative to the parental compound [93].

As part of the continuous development of the synthesis of potential antitumor derivatives of indole phytoalexins, the nucleoside analogs of 1-methoxybrassenin B, 1-(α-d-ribofuranosyl) brassenin B, and 1-(β-d-ribofuranosyl) brassenin B were suggested as well. The testing of the antiproliferative activity of these analogs on the Jurkat, CEM, CEM-VCR, MCF-7, and HeLa cancer cell lines revealed the significant activity of natural 1-methoxybrasenin B. The antiproliferative effect of individual nucleoside analogs were likely to decline with the loss of lipophilic features [94]. In general, indole nucleosides represent a rare type of natural products with interesting biological properties. Among them, the nucleoside rebeccamycin antibiotic and its analogs were identified as antineoplastic drugs. Through the glycosylation of natural indololcarbazole and its subsequent modifications, a potential anti-cancer drug J-107088 (edotecarin) was created showing an effect on MKN-45 cell (gastric cancer) implanted in mice. This substance belongs to the group of topoisomerase inhibitors [95,96,97].

Based on knowledge of the biological activity of indole phytoalexins, their isomers were obtained and tested as prospective anti-cancer substances. Regioisomer (isobrassinin (Figure 1)) showed the interesting antiproliferative effects of brassinin in cervical carcinoma, breast carcinoma, and epidermoid carcinoma cell lines. It inhibited from 70.7% to 89% of cell growth at a concentration of 30 μmol/L [98].

Enantiomeric forms of a 1-methoxyspirobrassinin and 2*R*,3*R*-(−)-1-methoxyspirobrassinol methyl ether were obtained through the spirocyclization method. The enantiomers of these indole phytoalexins were compared within the Jurkat, MCF-7, and HeLa tumor cell growth inhibition. The results of the study showed that a significant difference in antiproliferative activity among enantiomers occurred only with 1-methoxyspirobrassinol methyl ether, and only on the Jurkat cells. The concentration of 100 μmol/L of the 2*R*,3*R*-(−) form reduced the growth of these cells to 36.9% compared to the control, while the 2*S*,3*S*-(+) enantiomer at the same concentration slightly affected cell survival (79.8%). Other isomers showed a slight antiproliferative effect on all tested cell lines [99].

## 5. Conclusions

Research in the development and synthesis of new derivatives of indole phytoalexins which may have more favorable antiproliferative and chemopreventive properties than natural substances themselves is continuing. In the future, further experiments aimed at the elucidation of the mechanism of these noteworthy phytochemicals are necessary not only under in vitro but also in vivo conditions. The results of these studies have shown that these substances, due to their simple structure and antiproliferative activity, are potentially effective in developing new anti-tumor drugs that originate from nature.

## Figures and Tables

**Table 1:** Summarizing possible anti-cancer properties of representative indole phytoalexins and their derivatives.

Indole Phytoalexin	Possible Anti-Cancer Properties	Reference
Brassinin	Reduces the cell growth of mouse melanoma (B16) and leukemic cancer cell line (L1210)	[59]
	Exhibits cancer chemopreventive activity: inhibits the formation of preneoplastic mammary lesions in culture	[27]
	Induces phase II enzymes that metabolically inactivate chemical carcinogens	
	Enhances the effectiveness of tumor immunotherapy by blocking indoleamine 2,3-dioxygenase (IDO), the enzyme that drives immune escape in cancer	[65]
	Induces G1 phase arrest through increase of p21 and p27 by inhibition of the PI3K signaling pathway in human colon cancer cells (HT-29)	[67]
	Induces apoptosis in human prostate cancer cells (PC-3) through the suppression of PI3K/Akt/mTOR/S6K1 signaling cascades	[69]
	Inhibits STAT3 signaling through modulation of PIAS-3 and SOCS-3, thereby reducing tumor cell growthEnhances the antitumor effects of paclitaxel in human lung cancer xenograft in nude mice	[70]
	In combination with capsaicin, enhances apoptotic and anti-metastatic effects in human prostate cancer cells (PC-3)	[78]
	Potentiates vincristine cytotoxicity to U-87 MG (human glioblastoma astrocytoma)	[82]
Isobrassinin	Antiproliferative effect on cervical carcinoma (HeLa), breast carcinoma (MCF-7), and epidermoid carcinoma (A431) cell lines	[98]
5-Bromobrassinin	Suppresses growth of B16-F10 melanoma xenografts in C57BL/6 mice by inhibiting IDO enzyme	[28]
Homobrassinin	Induces mitotic phase arrest via inhibition of microtubule formation (dysregulation of α-tubulin, α1-tubulin, and β5-tubulin expression) in colorectal cancer cells (Caco-2)	[80]
	Induction of apoptosis in Caco-2 is associated with the loss of mitochondrial membrane potential, caspase-3 activation as well as intracellular reactive oxygen species (ROS) production.	
1-Methoxybrassinin	Exhibits antiproliferative effects on the human acute T lymphoblastic leukemia cell line (Jurkat) IC_50_ 10 μmol/L	[22]
	Induces apoptosis in Caco-2 cells, which is associated with the: - upregulation of pro-apoptotic genes expression (Bax) - downregulation of anti-apoptotic genes expression (Bcl-2) - activation of caspase-3,-7 - cleaveage of Poly (ADP-ribose) polymerase (*PARP*) - decrease intracellular GSH content	[24]
Cyclobrassinin	Exhibits antiproliferative effects on the epidermoid carcinoma cell line (KB) IC_50_ 8 μg/mL	[61]
	Exhibits cancer chemopreventive activity: inhibits the formation of preneoplastic mammary lesions in culture	[27]
	Induces phase II enzymes that metabolically inactivate chemical carcinogens	
Spirobrassinin	Reduces the cell growth of mouse melanoma (B16) and the leukemic cancer cell line (L1210)	[59]
	Exhibits cancer chemopreventive activity: inhibits the formation of preneoplastic mammary lesions in culture	[27]
	Induces phase II enzymes that metabolically inactivate chemical carcinogens	
	Potentiates vincristine cytotoxicity to U-87 MG (human glioblastoma astrocytoma)	[82]
	Reduces the growth of breast carcinoma cells (MCF-7, MDA-MB-231)	[19]
Camalexin	Antiproliferative activity on the human breast cancer cell line that overexpresses the Her2 (SKBr3) IC_50_ 2.7 μmol/L	[83]
	Increases expression of topoisomerase IIα in SKBr3	
	Induces apoptosis in prostate cancer cells (PCa) through the generation of ROS	
	Induces apoptosis in Jurkat cells by increasing production of ROS and activation of caspase-8 and caspase-9.	[85]
	Inhibits the growth of prostate cancer cells (PCa) by increasing activity of the cathepsin lysosomal enzyme (CD)	[86]
Benzocamalexin	The fusion of benzene to thiazole ring of camalexin significantly enhances its cytotoxicity	[23]
	In comparison with camalexin, significantly decreases survival of all tested cancer cell lines (IC_50_ ranging from 23.3 to 30.0 μmol/L)	
	Induces the mitotic phase arrest via inhibition of microtubule formation (downregulates the expression of α-tubulin, a1-tubulin, β5-tubulin) in Jurkat cells	
	Downregulates the expression of anti-apoptotic genes bcl-2, bcl-xL	
	Upregulates the expression of pro-apoptotic gene bax	
	Minimal toxicity (IC_50_ > 100.0 μmol/L) in non-cancer cells is observed	
